# Quality assessment of Clinical Practice Guidelines (CPG) for the diagnosis and treatment of inflammatory bowel disease using the AGREE II instrument: a systematic review

**DOI:** 10.1186/s12876-022-02539-9

**Published:** 2022-11-05

**Authors:** R. Zambrano-Sánchez, P. Alvarez-Mena, D. Hidalgo, C. M. Escobar Liquitay, J. V. A. Franco, R. W. M. Vernooij, D. Simancas-Racines, A. Viteri-García, C. Montesinos-Guevara

**Affiliations:** 1 Maestría en Epidemiología con mención en Investigación Clínica Aplicada. Facultad de Ciencias de la Salud Eugenio Espejo, Universidad UTE, Quito, Ecuador; 2 Internal medicine service, NMMC Hamilton, Hamilton, AL USA; 3 Research Department. Instituto Universitario Hospital Italiano de Buenos Aires, Buenos Aires, Argentina; 4 Institute of General Practice, Heinrich-Heine-University Düsseldorf, Düsseldorf, Germany; 5 Department of Nephrology and Hypertension, University Medical Center Utrecht, Utrecht University, Utrecht, The Netherlands. Julius Center for Health Sciences and Primary Care, University Medical Center Utrecht, Utrecht University, Utrecht, The Netherlands; 6 Centro de Investigación en Salud Pública y Epidemiologia Clínica (CISPEC), Facultad de Ciencias de la Salud Eugenio Espejo. Universidad UTE, Rumipamba and Bourgeois, Universidad UTE, 170147 Quito, Ecuador

**Keywords:** Inflammatory bowel disease, Crohn disease, Ulcerative colitis, Clinical practice guidelines, Systematic review

## Abstract

**Background:**

The incidence and diagnosis of inflammatory bowel disease (IBD) has increased considerably in recent years. Many clinical practice guidelines (CPG) have been developed for the management of this disease across different clinical contexts, however, little evidence exists on their methodological quality. Therefore, we aimed to systematically evaluate the quality of CPGs for the diagnosis and treatment of IBD using the Appraisal of Guidelines for Research and Evaluation (AGREE II) instrument.

**Methods:**

We identified CPGs by searching databases (MEDLINE - PubMed, EMBASE, CINAHL, LILACS) and other sources of gray literature on January 2022. We included guidelines with specific recommendations for the diagnosis and treatment of IBD and evaluated them with the AGREE II instrument to assess their methodological quality. Six independent reviewers assessed the quality of the guidelines and resolved conflicts by consensus. We assessed the degree of agreement using the intraclass correlation coefficient (ICC) and change in quality over time was appraised in two periods: from 2012 to 2017 and from 2018 to 2022.

**Results:**

We analyzed and evaluated 26 CPGs that met the inclusion criteria. The overall agreement among reviewers was moderate (ICC: 0.74; 95% CI 0.36 - 0.89). The mean scores of the AGREE II domains were: “Scope and purpose” 84.51%, “Stakeholder involvement” 60.90%, “Rigor of development” 69.95%, “Clarity of presentation” 85.58%, “Applicability” 26.60%, and “Editorial independence” 62.02%. No changes in quality were found over time.

**Conclusions:**

The quality of the CPGs evaluated was generally good, with a large majority of the assessed guidelines being “recommended” and “recommended with modifications”; despite this, there is still room for improvement, especially in terms of stakeholder involvement and applicability. Efforts to develop high quality CPGs for IBD need to be further optimized.

**Supplementary Information:**

The online version contains supplementary material available at 10.1186/s12876-022-02539-9.

## Introduction

Ulcerative colitis and Crohn’s disease are the main forms of inflammatory bowel disease (IBD). Both pathologies involve chronic inflammation of the gastrointestinal tract and show heterogeneity in terms of symptoms, which mainly include abdominal pain and diarrhea associated with malabsorption, weight loss and fever [1]. IBD involves periods of relapse and remission [2]. Although its etiology is unknown, it has been considered a multifactorial disease due to its association to genetic factors [3], immune mediators [4], changes in the intestinal microbiome [5] and exposure to various environmental agents [6].

The onset of IBD generally occurs around the third decade of life, but 25% of cases begin during childhood and adolescence [7]. The peak age of onset for Crohn’s disease is generally between 20 and 30 years of age, while Ulcerative Colitis usually begins at around 30 and 40 years of age [8].

The incidence and prevalence of IBD vary according to the geographic location, environment and ethnicity [9]. The latest reported data on the incidence of Ulcerative Colitis in North America and Europe ranged from 0 to 19.2 per 100,000 and 0.6 to 24.3 per 100,000, respectively [10]; whereas the prevalence of Ulcerative Colitis was 37.5 to 248.6 per 100,000 in North America and 4.9 to 505 per 100,000 in Europe [11]. For Crohn’s disease, the incidence varied from 0 to 20.2 per 100,000 in North America and from 0.3 to 12.7 per 100,000 in Europe [10]. In Latin America these data have considerable differences, however, in the last decades there has been a progressive increase with a prevalence of 0.99 to 44.3 per 100,000 inhabitants for Ulcerative Colitis and 0.24 to 16.7 per 100,000 inhabitants for Crohn’s disease [12, 13]. Epidemiological data suggest that the global incidence of IBD presents a marked increase, implying that the health systems of developing countries do not have the resources, health staff and infrastructure necessary for the diagnosis and treatment of the pathology.

Considering the increasing prevalence of IBD and its impacts in terms of health, society and economy (direct and indirect costs for the health systems and out-of-pocket expenses) [13], it is important to ensure high quality tools that facilitate its systematized treatment. For this reason, in the last decade, there have been important advances in terms of therapies for the management of IBD through pharmacological, non-pharmacological and surgical interventions [14, 15], these advances have been translated into several Clinical Practice Guidelines (CPG), which quality has not yet been assessed.

CPGs are systematically developed statements intended to help physicians and patients to make decisions about appropriate medical care in specific circumstances based on high-quality scientific evidence [16]. Their recommendations are intended to improve the quality of patient care by encouraging interventions of proven benefit and discouraging ineffective or potentially harmful interventions [16]. Several tools currently exist to assess the quality of a CPG and its implementation [17]; the AGREE (Appraisal of Guidelines, Research, and Evaluation) collaboration developed the AGREE II tool which is the most validated and widely used tool [18, 19]. This tool is helpful to assess the transparency in guidelines development and their quality, it provides a methodological strategy for guidelines development, and establishes a scheme for their reporting [20]. The AGREE II tool can be applied in Clinical Practice Guidelines (CPG) for diagnosis and medical interventions as well as for the evaluation of guidelines on health promotion, public health, among others [20].

Therefore, the main objective of this study is to systematically evaluate CPG for the diagnosis and treatment of IBD using the AGREE II tool, to provide evidence on their methodological quality and to assess changes in guideline quality over time.

## Methods

### Data Search

A systematic search was performed up to January 2022 to look for CPG on the diagnosis and treatment of IBD. CPGs were searched on databases (MEDLINE - PubMed, EMBASE, CINAHL, LILACS), professional societies (CAG, British Society of Gastroenterology, AGA, Brazilian Society of Gastroenterology), registries and guideline developers’ websites (NICE, SIGN). The full search strategy is detailed in Additional file 1*.*

### Inclusion and exclusion criteria

We included: 1.- CPGs with specific recommendations for the diagnosis and treatment of IBD, both for Crohn’s disease (CD) and ulcerative colitis (UC); 2.- CPGs on IBD that included pediatric, young, adult, and elderly populations; 3. - CPGs that provide the full search strategy that was conducted; 4.- CPGs that mentioned the process how they reached recommendations; 6.- CPGs published without date restriction until January 2022; and 6.- Last published available version of CPGs. The following documents were excluded: 1.- CPGs exclusively dealing with other clinical scenarios such as diagnostics (e.g. endoscopic, imaging), nutrition, immunological or surgical interventions for IBD; 2.- secondary publications (e.g., systematic reviews and meta-analyses) and 3.- abstracts from CPGs.

### Data Collection

Five reviewers working in pairs (DH, CMG, PA, RZ, RV) independently peer-screened the guidelines by title and abstract following the above inclusion and exclusion criteria. If the inclusion criteria were met, the full-text article were retrieved and screened by pairs for eligibility. All the screening process was performed using Rayyan (Rayyan Systems Inc) [21]. Two reviewers independently extracted the following data for each CPG: title, year of publication, submitting organization, type of funding, method used to collect evidence, number of sources documented, methods used to assess the quality and validity of the evidence, methods used to formulate the recommendations, country, and language. In case of disagreement, a third reviewer (VA, DSR) was involved.

### Quality assessment

The AGREE II instrument [18–20, 22] was used to evaluate the quality of the included CPGs. This instrument provides criteria for assessing the quality of the clinical practice guidelines through 23 items or questions, divided into 6 domains or categories; including: 1.- scope and purpose, 2.- stakeholder involvement, 3.- rigor of development, 4.- clarity of presentation, 5.- applicability, and 6.- editorial independence. The first domain evaluates the general objective of the CPG, specific health aspects and the target population; the second domain refers to the degree to which the guideline has been developed by the appropriate stakeholders and represents the views of intended users; the third domain refers to the process used to gather and synthesize evidence, the methods used to formulate and update recommendations; the fourth domain focuses on the language, structure and format of the guideline; the fifth domain refers to barriers and facilitators to CPG implementation, strategies for its adoption and resource considerations; and finally, the sixth domain is about the formulation of recommendations, to understand whether they are biased by conflicts of interest [19].

Each of the 23 items or questions is classified on a 7-point Likert-type scale, 7 being the maximum score corresponding to “strongly agree” and 1 the minimum score corresponding to “strongly disagree”.

For the global guideline evaluation, we used a 3-point scale: 1 “not recommended”, 2 “recommended with modifications” and 3 “recommended”. Six reviewers (DH, CMG, PA, RZ, JAF, RV), with clinical and methodological expertise, independently peer-scored each of the 23 items of the 6 domains of the AGREE II instrument for each CPG that was included. In case of disagreements with the assessment, a consensus was reached with the support of a third reviewer (AV, DSR).

### Statistical analysis

A descriptive analysis of the CPGs was performed using the general characteristics of each CPG from the extracted data. To calculate the score for each domain of the AGREE II tool, all item scores were summed up and the total value was standardized as a percentage of the maximum possible score for that domain, using the following formula:

$$\textrm{Standardized}\kern0.5em \textrm{score}\kern0.5em \left(\textrm{SP}\right)=\frac{\textrm{score}\kern0.5em \textrm{obtained}\kern0.5em \hbox{-} \kern0.5em \textrm{lowest}\kern0.5em \textrm{possible}\kern0.5em \textrm{score}}{\textrm{highest}\kern0.5em \textrm{possible}\kern0.5em \textrm{score}\kern0.5em \hbox{-} \kern0.5em \textrm{lowest}\kern0.5em \textrm{possible}\kern0.5em \textrm{score}}\times 100$$

With this method, the standardized score for each domain ranged from 0 to 100%. The result of the standardized score for each domain for all the guidelines is presented through the mean, median, first quartile (Q1), third quartile (Q3), interquartile range (IQR) and a boxplot. The degree of agreement between reviewers was assessed through the intraclass correlation coefficient (ICC) with a 95% confidence interval (CI). To visualize and compare the mean AGREE II scores obtained by the 26 CPGs assessed in this study, we generated a hexagonal radar graph where each domain is represented on a radial axis centered at 0 and the maximum score of each domain corresponds to each vertex of the hexagon. Finally, for the analysis of quality change over time, Student’s t-test was used to compare the means and categorize the CPGs into two periods: 2012 to 2017 and 2018 to 2022. Data analysis was performed in the statistical software RStudio v.1.4 [23] using the libraries ggplot2 [24], irr [25], tidyverse [26] and Table 1 [27].

**Table 1:** General characteristics of the CPGs

Guideline	Country	Organization	Year	Method used to asses quality and strength of evidence
AGA Clinical Practice Guidelines on the Management of Mild-to-Moderate Ulcerative Colitis [28]	USA	American Gastroenterological Association (AGA)	2019	GRADE^a^
ESPGHAN Revised Porto Criteria for the Diagnosis of Inflammatory Bowel Disease in Children and Adolescents [29]	UK	European Society of Pediatric Gastroenterology, Hepatology and Nutrition (ESPGHAN)	2013	Oxford Centre for Evidence-Based Medicine
ACG Clinical Guideline: Management of Crohn’s Disease in Adults [30]	USA	American College of Gastroenterology	2018	GRADE
European evidence based consensus on surgery for ulcerative colitis [31]	Multinational	European Crohn’s and Colitis Organization (ECCO)	2014	Oxford Center for Evidence-Based Medicine
Updated German Clinical Practice Guideline on “Diagnosis and treatment of Crohn’s disease” 2014 [32]	Germany	German Society for gastroenterology, digestive and metabolic diseases (DGVS) with the participation of Deutsche Gesellschaft for General and Visceral Surgery (DGAV), German Society of Surgery (DGCh), German Society for Internal Medicine (DGIM), German Society for Coloproctology (DGK), German Morbus Crohn’s / ulcerative colitis association (DCCV), Society for pediatric gastroenterology and nutrition (GPGE), Competence Network for Inflammatory Bowel Diseases.	2014	Oxford Center for Evidence-Based Medicine
Consensus guidelines of ECCO/ESPGHAN on the medical management of pediatric Crohn’s disease [33]	Multinational	European Crohn’s and Colitis Organization (ECCO / ESPGHAN)	2014	Oxford Center for Evidence-Based Medicine
Management of paediatric ulcerative colitis, Part 1: ambulatory care- an evidence-based guideline from ECCO and ESPGHAN [34]	Israel	Shaare Zedek Medical Center, The Hebrew University of Jerusalem, Israel.	2018	Oxford Centre for Evidence-Based Medicine
Evidence-based clinical practice guidelines for Crohn’s disease, integrated with formal consensus of experts in Japan [35]	Japan	Japanese Society of Gastroenterology and the Research group of Intractable Bowel Disease subsidized by the Ministry of the Health, Labour and Welfare of Japan	2013	Self-grading scheme used to assess the quality of the evidence
Diagnosis and treatment of inflammatory bowel disease: First Latin American Consensus of the Pan American Crohn’s and Colitis Organisation [36]	Mexico	Pan American Crohn’s and Colitis Organization	2016	Oxford Center for Evidence-Based Medicine
Mexican consensus for the diagnosis and treatment of idiopathic chronic ulcerative colitis [37]	Mexico	Mexican Association of Gastroenterology	2017	GRADE
Crohn’s disease Management in adults, children and young people [38]	UK	NICE National institute for health care and excellence	2012	GRADE
Second Korean guidelines for the management of ulcerative colitis [39]	Korea	Korean Association for the Study of Intestinal Diseases (KASID)	2017	GRADE
AGA Clinical Practice Guidelines on the Management of Moderate to Severe Ulcerative Colitis [40]	USA	AGA American Gastroenterological Association	2020	GRADE
Evidence-based clinical practice guidelines for inflammatory bowel disease [41]	Japan	The Japanese Society of Gastroenterology (JSGE)	2018	GRADE
Ulcerative colitis - treatment with biologicals [42]	Brazil	Brazilian Study Group on Inflammatory Bowel Disease, Brazilian Medical Association	2018	GRADE
British Society of Gastroenterology consensus guidelines on the management of inflammatory bowel disease in adults [43]	UK	British Society of Gastroenterology and others	2019	GRADE
Canadian Association of Gastroenterology Clinical Practice Guideline for the Medical Management of Pediatric Luminal Crohn’s Disease [44]	Canada	Canadian Association of Gastroenterology (CAG)	2019	GRADE
Clinical Practice Guideline for the Medical Management of Perianal Fistulizing Crohn’s Disease: The Toronto Consensus [45]	Canada	Canadian Association of Gastroenterology (CAG)	2018	GRADE
Crohn’s disease - treatment with biological medication [46]	Brazil	Brazilian Study Group on Inflammatory Bowel Disease, Brazilian Gastroenterology Federation, Brazilian Coloproctology Society, Brazilian Medical Association	2018	GRADE
Canadian Association of Gastroenterology Clinical Practice Guideline for the Management of Luminal Crohn’s Disease [47]	Canada	Canadian Association of Gastroenterology (CAG)	2019	GRADE
Evidence-based clinical practice guidelines for inflammatory bowel disease 2020 [48]	Japan	The Japanese Society of Gastroenterology (JSGE)	2021	GRADE
AGA Clinical Practice Guidelines on the Medical Management of Moderate to Severe Luminal and Perianal Fistulizing Crohn’s Disease [49]	USA	AGA American Gastroenterological Association	2021	GRADE
WSES-AAST guidelines: management of inflammatory bowel disease in the emergency setting [50]	Netherlands	The World Society of Emergency Surgery WSES	2021	GRADE
The Medical Management of Paediatric Crohn’s Disease: an ECCO-ESPGHAN Guideline Update [51]	Multinational	European Crohn’s and Colitis Organization [ECCO] and the Paediatric IBD Porto group of the European Society of Paediatric Gastroenterology, Hepatology and Nutrition [ESPGHAN]	2021	Oxford Center for Evidence-Based Medicine
Guidelines for the management of patients with Crohn’s disease. Recommendations of the Polish Society of Gastroenterology and the Polish National Consultant in Gastroenterology [52]	Poland	The Polish Society of Gastroenterology and the Polish National Consultant in Gastroenterology	2021	GRADE
ECCO Guidelines on Therapeutics in Crohn’s Disease: Medical Treatment [53]	Multinational	The European Crohn’s and Colitis Organization [ECCO]	2020	GRADE

^a^*GRADE* Grading of Recommendations, Assessment, Development and Evaluation

## Results

### Guideline characteristics

**Fig. 1:**
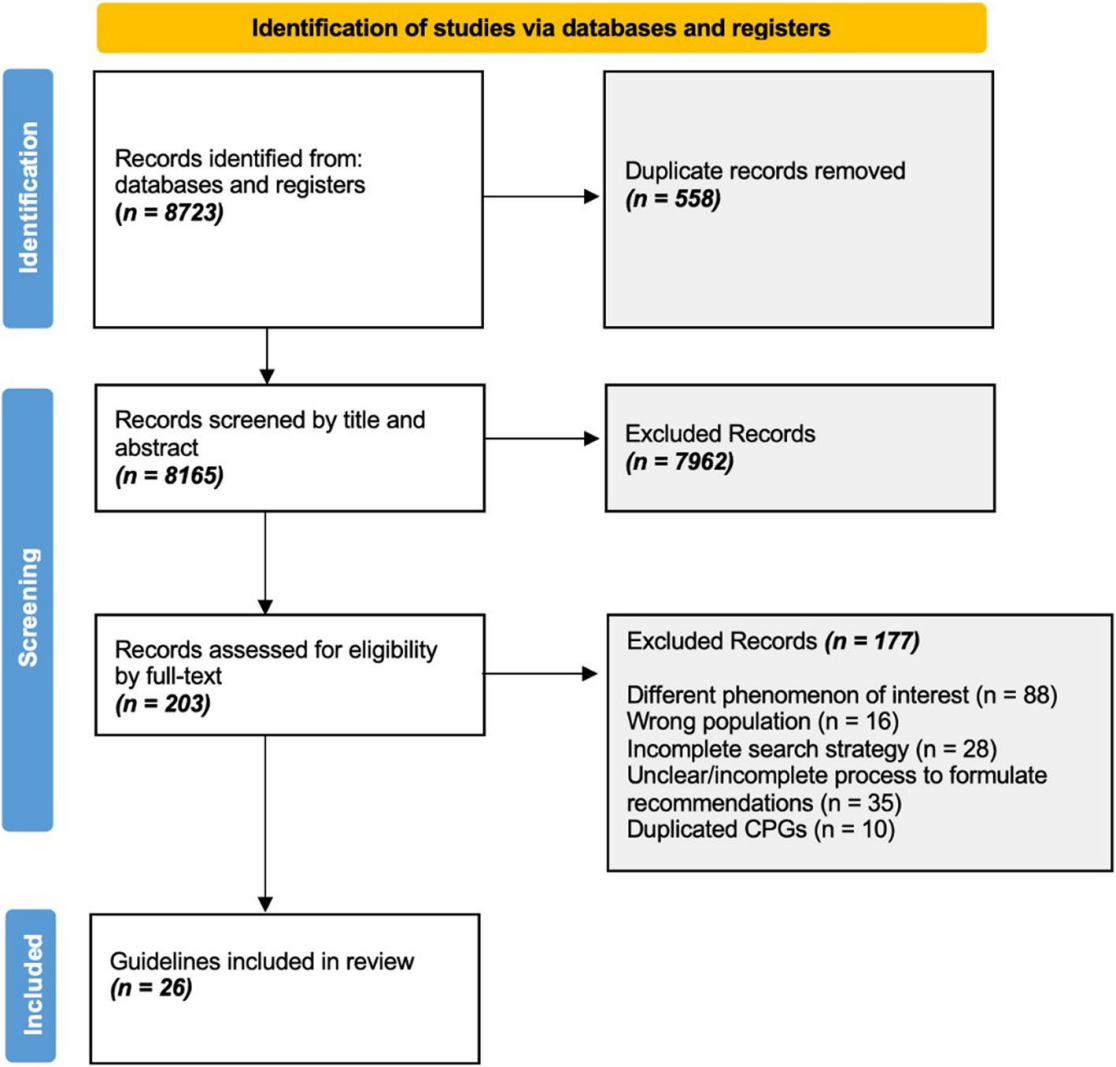
PRISMA flow diagram showing the flow of records that were obtained and reviewed throughout the different phases of the quality assessment

Eight thousand seven hundred twenty-three records were retrieved from the search strategy and 8165 remained after deduplication. 203 records were subsequently screened by full-text, of which 26 CPGs were included for data extraction after meeting the inclusion criteria (Fig. 1). Details on the characteristics of the included CPGs are shown in Table 1 [28–53].

Of the 26 included CPGs, four were from the United States (15.38%) and four were developed by an international collaboration (15.38%); three were from the United Kingdom, three from Canada and three from Japan (11.53% each), two were from Brazil and two from Mexico (7.69% each); one was from Germany, Israel, South Korea, the Netherlands and Poland (3.84% each). Included guidelines were published between 2012 and 2021 (see Table 1).

Three of the 26 guidelines focused exclusively on the pediatric population while the others were mainly focused on adults [29, 33, 51]. In terms of the scope of the CPGs, 22 dealt with diagnosis and clinical management [28–30, 32–41, 43–45, 47–49, 51–53], two with the use of biologic drugs only [42, 46], one with surgical management in the emergency setting [50] and one with the surgical management of ulcerative colitis [31]. All guidelines were considered evidence-based according to our a priori criteria.

Eighteen guidelines (69.23%) used the Grading of Recommendations Assessment, Development and Evaluation (GRADE) methodology to assess the quality of evidence and grade the strength of recommendations. Seven guidelines (26.92%) used the Oxford Centre for Evidence-Based Medicine criteria, and one guideline (3.84%) used a self-grading system to assess the quality of evidence (Table 1).

### Quality assessment

The agreement between the 6 reviewers was moderate with an ICC of 0.74 (95% CI: 0.36-0.89, p-value = 6.83e^−4^). A summary of the ICCs achieved by each pair of reviewers is shown in Table 2.

**Table 2:** Intraclass correlation coefficients (ICC) by peer reviewers

Pair of reviewers	ICC	95% CI	*P-value	^**a**^ICC interpretation
RZ + PA	0.69	0.02 - 0.90	0.020	Moderate
CM + JF	0.74	−0.07 - 0.97	0.065	Moderate
DH + RV	0.03	−0.03 - 0.74	0.292	Poor
DH + JF	0.69	−0.15 - 0.99	0.093	moderate
Global	0.74	0.36 - 0.89	6.83e^−4^	moderate

*Significance level < 0.05

^a^ICC interpretation following Ko and Li 2016 [53]

**Fig. 2:**
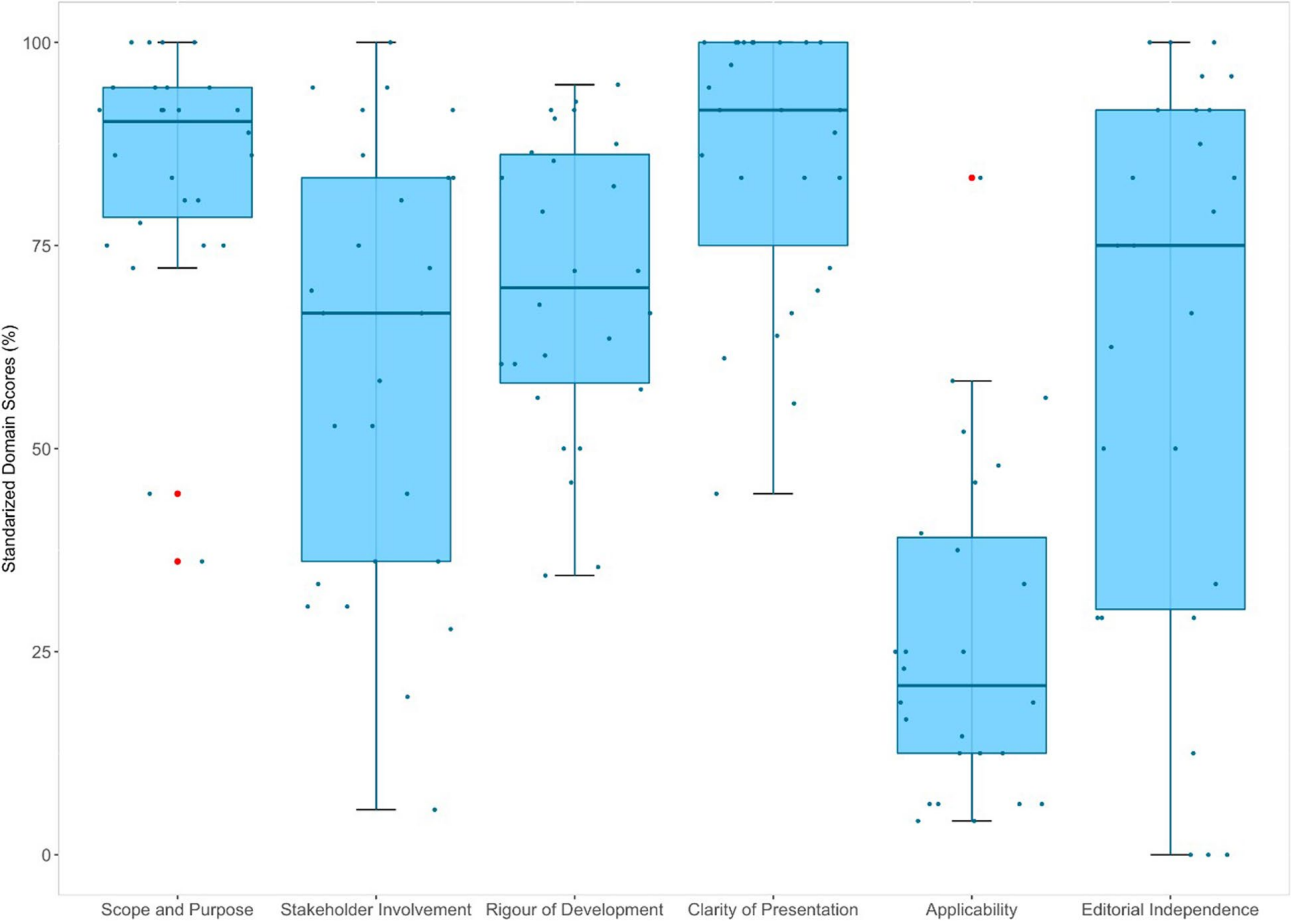
Distribution of standardized scores by domain for the 26 CPGs

Figure 2 shows a boxplot summarizing the statistical analysis of the standardized scores for each domain assessed with the AGREE II tool. In addition, Table 3 shows the standardized scores for all domain assessed in each clinical practice guideline.

**Table 3:** Standardized scores by domains of AGREE II

Guideline	Scope and Purpose	Stakeholder Involvement	Rigour of Development	Clarity of Presentation	Applicability	Editorial Independence	Overall Recommendation
AGA Clinical Practice Guidelines on the Management of Mild-to-Moderate Ulcerative Colitis [28]	94.44	30.56	60.42	83.33	25.00	75.00.	Recommended, with modifications
ESPGHAN Revised Porto Criteria for the Diagnosis of Inflammatory Bowel Disease in Children and Adolescents [29]	75.00	36.11	57.29	86.11	18.75	33.33	Recommended, with modifications
ACG Clinical Guideline: Management of Crohn’s Disease in Adults [30]	44.44	5.56	35.42	72.22	4.17	29.17	Not recommended
European evidence based consensus on surgery for ulcerative colitis [31]	75.00	36.11	50.00	66.67	6.25	29.17	Recommended, with modifications
Updated German Clinical Practice Guideline on “Diagnosis and treatment of Crohn’s disease” 2014 [32]	91.67	86.11	83.33	61.11	12.50	91.67	Recommended, with modifications
Consensus guidelines of ECCO/ESPGHAN on the medical management of pediatric Crohn’s disease [33]	72.22	52.78	61.46	83.33	12.50	12.50	Recommended, with modifications
Management of paediatric ulcerative colitis, Part 1: ambulatory care- an evidence-based guideline from ECCO and ESPGHAN [34]	83.33	27.78	67.71	83.33	6.25	66.67	Recommended, with modifications
Evidence-based clinical practice guidelines for Crohn’s disease, integrated with formal consensus of experts in Japan [35]	91.67	52.78	56.25	94.44	16.67	79.17	Recommended, with modifications
Diagnosis and treatment of inflammatory bowel disease: First Latin American Consensus of the Pan American Crohn’s and Colitis Organisation [36]	75.00	30.56	60.42	69.44	12.50	50.00	Recommended, with modifications
Mexican consensus for the diagnosis and treatment of idiopathic chronic ulcerative colitis [37]	36.11	19.44	50.00	63.89	6.25	62.50	Not recommended
Crohn’s disease Management in adults, children and young people [38]	94.44	83.33	94.79	100.00	83.33	75.00	Recommended
Second Korean guidelines for the management of ulcerative colitis [39]	80.55	58.33	71.88	100.00	22.92	29.17	Recommended, with modifications
AGA Clinical Practice Guidelines on the Management of Moderate to Severe Ulcerative Colitis [40]	100.00	80.56	91.67	100.00	56.25	83.33	Recommended
Evidence-based clinical practice guidelines for inflammatory bowel disease [41]	91.66	75.00	82.29	88.89	58.33	83.33	Recommended, with modifications
Ulcerative colitis - treatment with biologicals [42]	88.88	33.33	45.83	55.56	6.25	0.00	Not recommended
British Society of Gastroenterology consensus guidelines on the management of inflammatory bowel disease in adults [43]	100.00	100.00	91.67	100.00	45.83	100.00	Recommended
Canadian Association of Gastroenterology Clinical Practice Guideline for the Medical Management of Pediatric Luminal Crohn’s Disease [44]	100.00	94.44	85.42	100.00	25.00	95.83	Recommended
Clinical Practice Guideline for the Medical Management of Perianal Fistulizing Crohn’s Disease: The Toronto Consensus [45]	86.11	91.67	79.17	100.00	14.58	95.83	Recommended, with modifications
Crohn’s disease - treatment with biological medication [46]	80.55	44.44	34.38	44.44	4.17	0.00	Not recommended
Canadian Association of Gastroenterology Clinical Practice Guideline for the Management of Luminal Crohn’s Disease [47]	100.00	91.67	90.63	100.00	37.50	91.67	Recommended
Evidence-based clinical practice guidelines for inflammatory bowel disease 2020 [48]	77.78	66.67	66.67	91.67	18.75	87.50	Recommended, with modifications
AGA Clinical Practice Guidelines on the Medical Management of Moderate to Severe Luminal and Perianal Fistulizing Crohn’s Disease [49]	94.44	83.33	87.50	100.00	52.08	91.67	Recommended
WSES-AAST guidelines: management of inflammatory bowel disease in the emergency setting [50]	86.11	69.44	63.54	91.67	25.00	50.00	Recommended, with modifications
The Medical Management of Paediatric Crohn’s Disease: an ECCO-ESPGHAN Guideline Update [51]	91.67	66.67	86.46	100.00	39.58	100.00	Recommended, with modifications
Guidelines for the management of patients with Crohn’s disease. Recommendations of the Polish Society of Gastroenterology and the Polish National Consultant in Gastroenterology [52]	94.44	72.22	71.88	91.67	33.33	0.00	Recommended, with modifications
ECCO Guidelines on Therapeutics in Crohn’s Disease: Medical Treatment [53]	91.67	94.44	92.71	97.22	47.92	100.00	Recommended
**Mean Score**	84.51	60.90	69.95	85.58	26.60	62.02	
**Median**	90.27	66.67	69.79	91.67	20.83	75.00	

#### Domain 1: Scope and purpose

This domain evaluates the general objective of the CPG, specific health aspects and the target population [19]. The mean score was 84.51% (median: 90.27%, Q1: 78.47%, Q3: 94.44% and IQR = 15.97%; Fig. 2). Twenty-four CPGs (92.30%) scored above 60% in this domain [28, 29, 31–36, 38–53]. See Table 3 for details on domain 1.

#### Domain 2: Stakeholder involvement

This domain refers to the degree to which the guideline has been developed by the appropriate stakeholders and represents the views of intended users [19]. The mean score was 60.90% (median: 66.67%, Q1: 36.11%, Q3: 83.33% and IQR = 47.22%; Fig. 2). Fourteen CPGs (53.84%) scored above 60% in this domain [32, 38, 40, 41, 43–45, 47–53]. See Table 3 for details on domain 2.

#### Domain 3: Rigor of development

This domain refers to the process used to gather and synthesize evidence, the methods used to formulate and update recommendations [19]. The mean score was 69.95% (median: 69.79%, Q1: 58.07%, Q3: 86.20% and IQR = 28.12%; Fig. 2). Nineteen CPGs (73.07%) scored above 60% in this domain [28, 32–34, 36, 38–41, 43–45, 47–53]. See Table 3 for details on domain 3.

#### Domain 4: Clarity of presentation

This domain focuses on the language, structure and format of the guideline [19]. The mean score was 85.58% (median: 91.67%, Q1: 75.00%, Q3: 100.00% and IQR = 25%; Fig. 2). Twenty-four CPGs (92.30%) scored above 60% in this domain [28–41, 43–45, 47–53]. See Table 3 for details on domain 4.

#### Domain 5: Applicability

This domain refers to barriers and facilitators to CPG implementation, strategies for its adoption and resource considerations [19]. The mean score was 26.60% (median: 20.83%, Q1: 12.50%, Q3: 39.06% and IQR = 26.56%; Fig. 2). Only one CPG (3.84%) scored above 60% in this domain [38]. See Table 3 for details on domain 5.

#### Domain 6: Editorial Independence

This domain is about the formulation of recommendations, understand whether they are biased by conflicts of interest [19]. The mean score was 62.02% (median: 75.00%, Q1: 30.21%, Q3: 91.67% and IQR = 61.45%; Fig. 2). Sixteen CPGs (61.53%) scored above 60% in this domain [28, 32, 34, 35, 37, 38, 40, 41, 43–45, 47–49, 51, 53]. See Table 3 for details on domain 6.

#### Overall assessment

Seven out of the 26 evaluated CPGs (26.9%) were “recommended” by the independent reviewers [38, 40, 43, 44, 47, 49, 53]. Most of the CPGs, 15 guidelines (57.7%), were “recommended with modifications” [28, 29, 31–36, 39, 41, 45, 48, 50–52]. Finally, 4 CPGs (15.4%) were “not recommended” (see Table 3) [30, 37, 42, 46].

#### Combined assessment

**Fig. 3:**
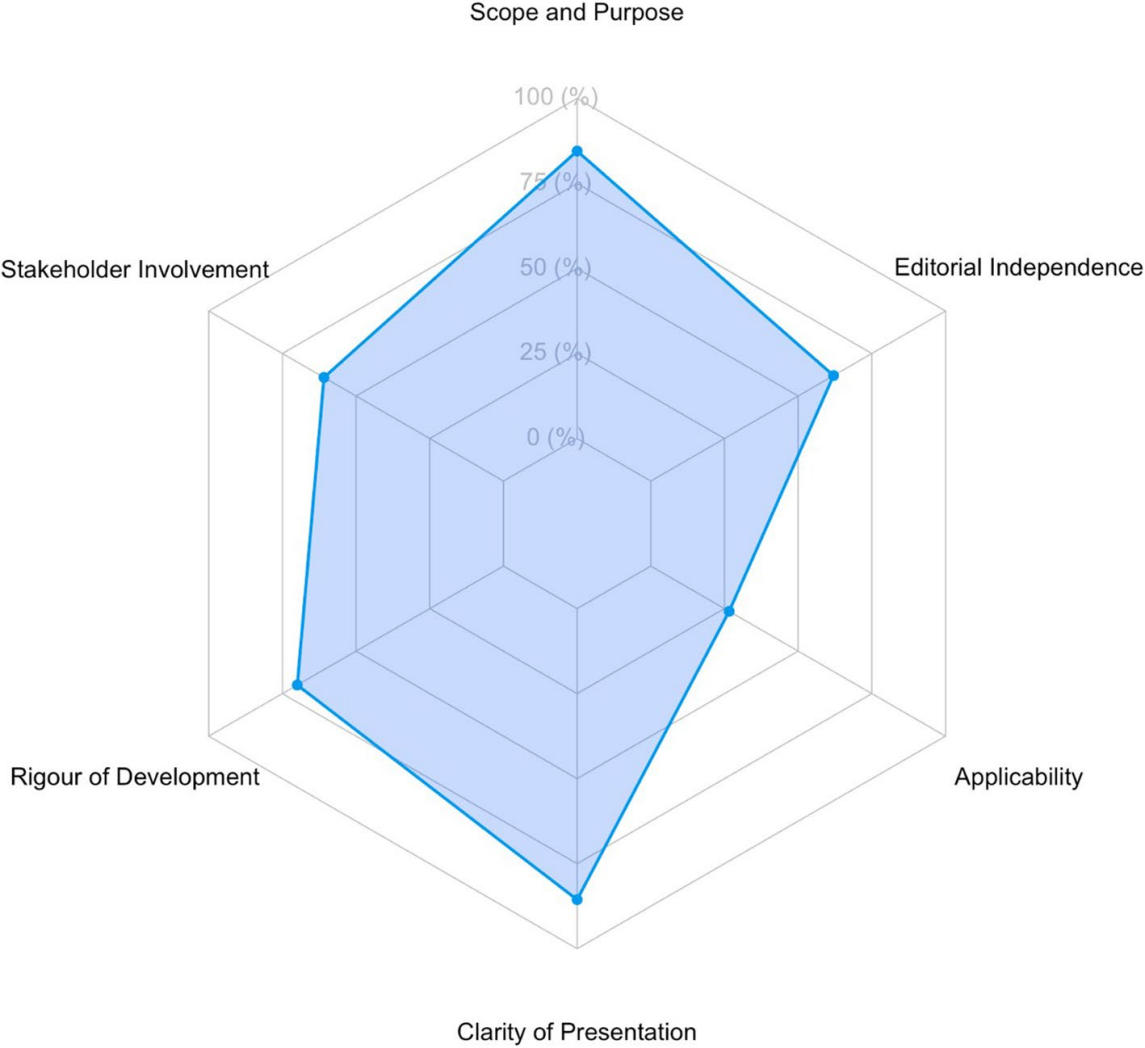
Radar chart of the mean standardized scores by domains of the 26 IBD CPGs assessed

Finally, in the radar plot analysis we observe that domains “scope and purpose”, “stakeholder involvement”, “rigor of development”, “clarity of presentation” and “editorial independence” show similar areas in the scores achieved; however, the domain “applicability” is notoriously deficient in all the evaluated guidelines (Fig. 3).

### Quality assessment over time

With respect to quality change over time, no statistically significant differences were found for the means of the standardized scores for each AGREE II domain between the guidelines published during the 2012-2017 period and those published between 2018 and 2022 (Table 4).

**Table 4:** Quality changes over time

Domain	Guidelines from 2012-2017	Guidelines from 2018-2022	**P-value
Scope and purpose	81.9 (9.15)	85.6 (17.9)	0.5868
Stakeholder involvement	54.5 (21.0)	63.7 (29.4)	0.4336
Rigour of development	66.9 (15.3)	71.3 (19.6)	0.5817
Clarity of presentation	82.6 (15.3)	86.9 (17.0)	0.5521
Applicability	23.2 (24.8)	28.1 (19.0)	0.5815
Editorial independence	50.0 (28.7)	67.4 (36.3)	0.2444

*Data given as mean +/− (SD) of standardized scores

**Significance level < 0.05, p-value with Student’s t method for the difference of two means

## Discussion

### What do the findings of this study mean?

This review showed that the evaluated IBD CPGs had an acceptable quality based on the AGREE II instrument since 7 out of the 26 evaluated guidelines were “recommended”, 15 were “recommended with modifications” and only 4 were “not recommended”. The domains with the highest scores were “clarity of presentation” and “scope and purpose”, which reached values over 60%, indicating that most of the assessed guidelines had well-defined general and specific objectives, the population to which the guideline was intended to apply was well defined, and the recommendations were clearly described and identifiable. Rigor of development was the domain that received the third best score with 69.95%; this domain could be argued to have the greatest effect on the quality of a clinical practice guideline, since it has to do with the entire process used to formulate and construct the recommendations and it is the one that comprises the most items within AGREE II for its evaluation [54]. We consider that a score over 60% is more than acceptable for “rigor of development”, which achieved this score due to most guidelines were partly penalized for being unclear with the description of external experts’ assessment and for not having an explicit updating statement.

The domains “stakeholder involvement” and “editorial independence” obtained scores slightly over 60% (60.90 and 62.02%, respectively; Fig. 3), which indicates that the views and preferences of patients still need to be considered when the CPG is drafted and that an expert methodologist/epidemiologist should be included in the guideline drafting group. In addition, both domains achieved low scores due to the limited information most guidelines provided in terms of funding and its influence on the guidelines’ content, as well as the lack of detail they included regarding conflicts of interest and how these conflicts were dealt. Considering these limitations on the development of CPGs could contribute to their improvement.

The “applicability” domain was the worst scored domain in this review with an average score of 26.60% (Fig. 3), well below the 60% cut-off point for this domain. The main reason for this is that most guideline developers do not fully consider guideline’s implementation in terms of facilitators and barriers for guidelines’ applicability or they do not fully consider the resources and tools that are available in a specific context. We also noted that most of the guidelines did not consider the economic impact of their recommendations on resources and health budgets, for example, most guidelines did not include health economists in the guideline development group or did not perform cost-benefit analysis. The limitations and omissions that have been observed in the included guidelines restraint the translation of these documents into clinical practice, thus hindering its operability.

Regarding quality change over time, this study failed to demonstrate statistically significant differences between guidelines published during the 2012-2017 period versus guidelines published between 2018 and 2022 (see Table 4) for any domain covered by AGREE II. This finding may be due to the small sample size in this study, which is associated to the specific inclusion criteria applied in the selection of CPGs as well as the large variety of CPGs for IBD (clinical, surgical, preventive, etc.) we encountered when screening. In addition, the time ranges we compared were too short since guidelines’ development in terms of IBD and our study’s criteria has been an early activity. However, one point to highlight is the implementation and dissemination of the GRADE methodology in the development of guidelines, especially in those produced in the last 4 years; our study found that 17 out of the 26 included CPGs had used this methodology as a framework for grading the evidence and formulating their recommendations.

### The context of this review with other literature

While this review is not the first to evaluate clinical practice guidelines on inflammatory bowel disease, it is the first to evaluate a large sample of CPGs as there was no date restriction in its search, which gave us a much broader picture of what has been produced in the past and current time. Thus, in line with other reviews of CPG for IBD conducted by other investigators, and addressing different contexts of inflammatory bowel disease, the domains with the highest scores were “clarity of presentation” and “scope and purpose” and the domains with the lowest scores were “stakeholder involvement” and “applicability” [55–57]. These results are also similar to previous CPG evaluations for other clinical-surgical areas such as interventional radiology, pediatrics or dermatology [58–60].

In addition, other studies that investigated quality changes over time for clinical practice guidelines in other specialties did not find evidence of significant changes in quality in the different evaluated periods of time [61–63]. These results are consistent with the findings of this study. However, studies by Bhatt et al. [64] for pediatric type II diabetes CPG and Acuña-Izcaray et al. [65] for asthma CPG, found statistically significant differences in quality over time for the selected periods for each individual domain, while a statistical significance has not been found for all domains at the same time.

### Strengths and limitations

Although a strength of our systematic review was the broad and exhaustive approach of our search – carried out in databases, compiling entities and guideline developers, with a sensitive strategy designed for this purpose – it is possible that our review may have missed some CPGs that were not adequately indexed or that dealt with other contexts related to inflammatory bowel disease. Likewise, our study only included CPGs published in English or Spanish, factors that could have contributed to a potential selection bias.

Likewise, having chosen CPGs with well-defined inclusion criteria, it is likely that our results have overestimated the score obtained by selecting guidelines that would score higher than the entire possible universe of CPGs for IBD. Therefore, our conclusions acquire more relevance when evaluating this type of guidelines.

On the other hand, although the degree of agreement reached by the reviewers was moderate (ICC = 0.74), this may be due to the fact that the AGREE II instrument weights each item with a 7-point Likert-type scale, where only the extreme values of this scale are well defined, but it is prone to subjectivity for intermediate values 3, 4 and 5 on the scale. As our research had a large number of reviewers (six), reaching a higher value for the intraclass correlation coefficient (ICC) to improve reliability was difficult. However, we consider that the value achieved does provide adequate reliability [66].

In addition, since the implementation of the AGREE II tool in 2010, it has become the most widely used and popular resource for assessing the quality of CPGs, choosing a cut-off point above which a guideline can be defined as having good quality is subjective and this selection will depend on the context in which the review is being performed. As Brouwers et al. [67] noted, “there is no evidence that if a guideline exceeds a certain score, the recommendations are easier to adopt, or improve processes of care, or lead to better patient outcomes than guidelines that do not achieve that score”.^(^ [67]^, p.195)^ That is, the validity of the overall assessment may be limited, as there are no clear rules yet on how to weigh the different domain scores to make a decision on whether or not to recommend guidelines.

### What is new and conclusion

Overall, this study determined that the quality of clinical practice guidelines for the diagnosis and treatment of inflammatory bowel disease is acceptable and that there is still room for improvement, especially in terms of stakeholder participation (inclusion of patients, expert methodologists/epidemiologists) and applicability (enablers, barriers, optimization of resources, external review). It is desirable that guideline developers consider these shortcomings in the future for the overall improvement of guidelines’ quality to reduce clinical practice heterogeneity in IBD.

## Supplementary Information

**Additional file 1.**

## Data Availability

The datasets used and/or analysed during the current study are available from the corresponding author on reasonable request.

## Abbreviations

CPG: Clinical practice guideline
AGREE: Appraisal of Guidelines, Research, and Evaluation
GRADE: Grading of Recommendations, Assessment, Development and Evaluation
NPRISMA: Preferred Reporting Items for Systematic Reviews and Meta-Analyses
IBD: Inflammatory bowel disease
CD: Crohn’s disease
UC: Ulcerative colitis
ICC: Intraclass correlation coefficient
IQR: Interquartile range
SD: Standard deviation
Qi: Quartile
CI: Confidence interval
