# Supplementary material for: Quality assessment of Clinical Practice Guidelines (CPG) for the diagnosis and treatment of inflammatory bowel disease using the AGREE II instrument: a systematic review
Source: BMC Gastroenterol. 2022 Nov 5;22:447. doi: 10.1186/s12876-022-02539-9 (PMC9637309; doi:10.1186/s12876-022-02539-9)
Supplement: Supplementary file 1 — Additional file 1. [file 12876_2022_2539_MOESM1_ESM.docx]

**Additional file 1.**

**Health science databases**

**Medline** (<https://pubmed.gov/>)

**#1:** "Inflammatory Bowel Diseases" [Mesh]

**#2:** "Crohn Disease" [Mesh]

**#3:** "Colitis, Ulcerative" [Mesh]

**#4:** inflammatory bowel diseases [Title/Abstract]

**#5:** regional enteriti* [Title/Abstract]

**#6:** ileocoliti* [Title/Abstract]

**#7:** terminal ileiti* [Title/Abstract]

**#8:** regional ileiti* [Title/Abstract]

**#9:** idiopathic* proctocolitis [Title/Abstract]

**#10:** colitis ulcerative [Title/Abstract]

**#11:** primary sclerosis cholangitis [Title/Abstract]

**#12:** IBD[Title/Abstract]

**#13:** UC[Title/Abstract]

**#14:** CD[Title/Abstract]

**#15:** #1 OR #2 OR #3 OR #4 OR #5 OR #6 OR #7 OR #8 OR #9 OR #10 OR #11 OR #12 OR #13 OR #14

**#16:** "guideline" [Publication Type]

**#17:** "Practice Guideline" [Publication Type]

**#18:** Clinical practice guide [Title/Abstract]

**#19:** Practice Guideline [Title/Abstract]

**#20:** #16 OR #17 OR #18 OR #19

**#21:** #15 AND #20

**Embase (Elsevier.com)**

**#1:** 'inflammatory bowel disease'/exp

**#2:** 'crohn disease'/exp

**#3:** 'ulcerative colitis'/exp

**#4:** 'inflammatory bowel diseases':ab,ti

**#5:** 'regional enteriti*':ab,ti

**#6:** ileocoliti*:ti,ab

**#7:** 'terminal ileiti*':ab,ti

**#8:** 'regional ileiti*':ab,ti

**#9:** 'idiopathic* protocolitis':ab,ti

**#10:** 'ulcerative colitis':ti,ab

**#11:** 'primary sclerosis colangitis':ab,ti

**#12:** ibd:ab,ti

**#13:** cd:ab,ti

**#14:** uc:ab,ti

**#15:** #1 OR #2 OR #3 OR #4 OR #5 OR #6 OR #7 OR #8 OR

#9 OR #10 OR #11 OR #12 OR #13 OR #14

**#16:** 'practice guideline'/exp

**#17:** guideline:ab,ti

**#18:** 'clinical practice guide':ab,ti

**#19:** #16 OR #17 OR #18

**#20:** #15 AND #19

**LILACS** (<https://lilacs.bvsalud.org>)

(tw:((guía OR guideline) OR (guía de prática clínica OR  practice guideline))) AND (tw:((regional ileitis OR ileíte regional))) OR (tw:((idiopathic proctocolitis OR proctocolitis idiopática OR proctocolite idiopática))) OR (tw:((colite ulcerativa OR colitis ulcerative OR colitis ulcerosa))) OR (tw:((ileítis terminal OR terminal de ileíte OR ileitis terminal))) OR (tw:((ileocolite OR ileocolitis))) OR (tw:((enteritis regional OR regional enteritis OR enterite regional))) OR (tw:((colitis ulcerosa  OR colitis, ulcerative OR colite ulcerativa))) OR (tw:((doença de crohn  OR crohn disease OR  enfermedad de crohn))) OR (tw:((doenças inflamatórias intestinais  OR inflammatory bowel diseases  OR enfermedades inflamatorias del intestino)))) AND mj:("Guías de Práctica Clínica como Asunto")

**CINAHL Database | EBSCO** (<https://www.ebsco.com>)

((Inflammatory Bowel Diseases OR Colitis Ulcerative OR Regional Enteritis OR ileocolitis OR Crohn Disease OR Terminal de Ileíte OR Idiopathic* proctocolitis OR Colitis ulcerative)) AND ((Guideline OR Clinical practice Guide OR Practice Guideline))

**Organizations and other websites (professional societies, registries and guideline developers´ websites)**

1. The National Institute for Health and Care Excellence (NICE); [www.nice.org.uk](http://www.nice.org.uk)
2. Scottish Intercollegiate Guidelines Network (SIGN); [www.sign.ac.uk](http://www.sign.ac.uk)
3. American College of gastroenterology (ACG); <https://gi.org>
4. Guidelines International Network (GIN); <https://g-i-n.net>
5. American Gastroenterological association (AGA); <https://gastro.org>
6. Portuguese Gastronterology society; <https://www.spg.pt/portuguese-society-of-gastroenterology/>
7. Gastroenterological society of Australia; <https://www.gesa.org.au>
